# A symbiotic liaison between the genetic and epigenetic code

**DOI:** 10.3389/fgene.2014.00113

**Published:** 2014-05-01

**Authors:** Holger Heyn

**Affiliations:** Cancer Epigenetics and Biology Program, Bellvitge Biomedical Research InstituteBarcelona, Spain

**Keywords:** DNA methylation quantitaive trait loci, DNA methylation, GWAS, EWAS, epigenetic regulation

## Abstract

With rapid advances in sequencing technologies, we are undergoing a paradigm shift from hypothesis- to data-driven research. Genome-wide profiling efforts have given informative insights into biological processes; however, considering the wealth of variation, the major challenge still remains in their meaningful interpretation. In particular sequence variation in non-coding contexts is often challenging to interpret. Here, data integration approaches for the identification of functional genetic variability represent a possible solution. Exemplary, functional linkage analysis integrating genotype and expression data determined regulatory quantitative trait loci and proposed causal relationships. In addition to gene expression, epigenetic regulation and specifically DNA methylation was established as highly valuable surrogate mark for functional variance of the genetic code. Epigenetic modification has served as powerful mediator trait to elucidate mechanisms forming phenotypes in health and disease. Particularly, integrative studies of genetic and DNA methylation data have been able to guide interpretation strategies of risk genotypes, but also proved their value for physiological traits, such as natural human variation and aging. This *Review* seeks to illustrate the power of data integration in the genomic era exemplified by DNA methylation quantitative trait loci. However, the model is further extendable to virtually all traceable molecular traits.

## INTRODUCTION

Owing to the boost in detection technologies, numerous genetic and epigenetic variations have been related to phenotypic variability, including human diseases. Their interpretation has given an informative insight into aberrant biological processes and has led to the identification of novel targets for therapeutic interventions (Garraway and Lander, 2013). However, considering the wealth of alterations detectable by genome-wide studies, the major challenge still remains in the discrimination between driving events and those that are functionally silent or mere consequence of the disease. Furthermore, alterations frequently occur in a non-coding context, complicating their functional interpretation (Hindorff et al., 2011).

The comprehensive landmarking of functional elements in the human genome provided a measure of regional transcriptional activity and regulatory potential (Consortium, 2012). Latter regions were categorized by representative histone marks, DNA methylation levels, and chromatin conformations. However, their annotation does not immediately provide functional insights, as most of regulatory loci cannot be clearly assigned to target genes, especially those outside the promoter context. Nevertheless, various studies reported a significant overlap between phenotype-associated polymorphisms with regulatory histone marks (Hnisz et al., 2013), differentially methylated regions (Ziller et al., 2013) or open chromatin formations (Paul et al., 2013), suggesting their implication in functional downstream cascades and phenotype formation.

However, despite the comprehensive functional annotation of the human genome, we largely lack mechanistic interpretations for the majority of genetic variations and genotype–phenotype associations. To improve our understanding of causal relationships, we need to undergo a paradigm shift from analyzing loci function to loci effects; and shifting from annotation toward functional linkage studies. These are in particular straightforward to interpret if a component of the associations can be directly connected to gene function or activity. An illustrative and well-established example of functional linkage analysis is represented by expression quantitative trait loci (eQTL), wherein *cis*- or *trans*-located genetic polymorphisms present significant associations to gene activity, determined by transcript abundance and even transcript variants (Grundberg et al., 2012; Lappalainen et al., 2013). The identification of direct impacts of polymorphisms on transcriptional activity suggests causality, with the genetic variant and altered transcription being the cause and consequence, respectively. Particularly valuable for intergenic and intronic polymorphisms that are otherwise challenging to interpret, eQTL directly point to gene targets and hence facilitate the identification of disease driving mechanisms (Li et al., 2010, 2013).

## EPIGENETIC MEDIATOR FUNCTION IN GENE REGULATION

Functionally, the linkage model can be further extended including mediator traits that regulate gene expression. In this regard, virtually all regulatory events can be assigned as mediator, including histone modifications, DNA methylation, non-coding RNAs, or chromatin structure. Intriguingly, although the intense interplay between different layers of epigenetic regulation is accepted knowledge (Bernstein et al., 2012), it is poorly understood how the genetic and epigenetic code interact (Schübeler, 2012).

In this respect, computational strategies linking the genome to the epigenome, suggest a profound intrinsic association between epigenetic marks and genetic sequence, with cumulative effects of many weak interactions eventually forming the epigenetic code (Yuan, 2012). Consistently, to a certain extend epigenetic landscapes could be predicted *in silico* and assembled *in vitro,* suggesting the epigenome to be partly encoded in the genome (Kaplan et al., 2009). Herein, specifically DNA methylation patterns present strong genetic associations (Weber et al., 2005; Lienert et al., 2011). However, the genetic blueprint is likely to permit certain epigenetic states without precisely defining shape or timing. Certainly, the linkage between genetic variance and gene regulation could improve our knowledge about phenotype biology and the underlying mechanistic chain of events. Furthermore, an integrative analysis of genetic variation with the gene regulome could facilitate the interpretation of non-coding genetic variance and could further support strategies to reliably identify phenotype driving events.

Technically, it is important to distinguish between two concepts of functional linkage studies: the analysis of overlapping coordinates, defining direct mechanistic effects (Kasowski et al., 2010; McDaniell et al., 2010) and the definition *cis*-acting connections of distal elements, suggesting their regulatory relationships (Gibbs et al., 2010; Zhang et al., 2010). While the first is identifying additional layers of regulation controlling the activity of a specific locus (possibly being directly affected by the genetic variation; McDaniell et al., 2010; Boyle et al., 2012; Schaub et al., 2012; Paul et al., 2013), the latter is defining a sequential chain of events, strongly suggesting functional importance. This *Review* will focus on the relationship between *cis*-associated distal elements, as particularly these interactions provide superior information compared to on-site variation. Indeed, *cis*-associations not only define local activity, but determine functionality in downstream processes.

Despite the wealth of regulatory mechanisms, this *Review* focuses on DNA methylation, as the covalent modification of cytosines in a CpG context presents a stable and reliably detectable epigenetic mark with high impact on gene regulation (Jones, 2012). Generally, the DNA hypermethylation, in particular in promoter regions, was associated to transcriptional silencing, whereas promoter hypomethylation favors gene expression. In contrary, the DNA methylation levels in the gene body often correlate positively with transcriptional activity (Jones, 2012; Kulis et al., 2012). Importantly from an integrative perspective, DNA methylation levels were shown to be partially dependant on the underlying genetic sequence defined as DNA methylation quantitative trait loci (meQTL; Kerkel et al., 2008; Shoemaker et al., 2010). Herein, CpG methylation levels present high correlations with the genotype of non-overlapping nucleotides. Specifically, DNA methylation levels appear to segregate with genotypes in their proximity (*cis*-acting) rather than being farther distributed, suggesting the contribution of the local genomic environment to establish the connections (Shoemaker et al., 2010; Grundberg et al., 2013; Heyn et al., 2013). Although specific mechanisms establishing the association between the genetic and epigenetic code are widely unknown, their identification suggested causal connections between genetic and epigenetic information. From a genomic perspective, meQTL present a powerful surrogate mark to elucidate functional and mechanistic implementations of phenotype-associated genetic variations.

## A CASCADE OF GENETIC AND EPIGENETIC REGULATION

**FIGURE 1:**
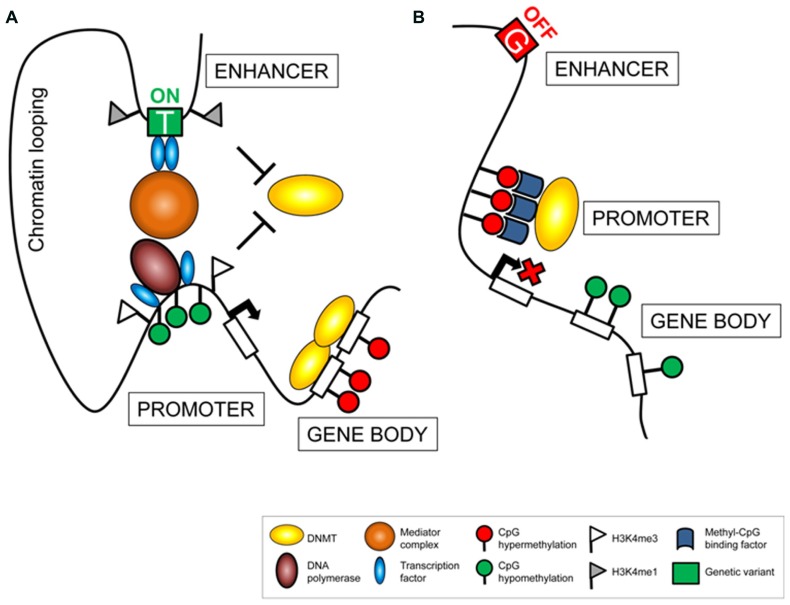
**Genetic variance affects the regulatory machinery**. Polymorphic alleles located in *cis*-regulating elements (e.g., enhancer sites) can be associated to variation in downstream cascades resulting in transcriptional activation **(A)** or silencing **(B)**. Changes in gene regulation can be assessed measuring variance in gene expression and related regulatory factors, such as epigenetic modifications (e.g., DNA methylation) or occupancy of regulatory proteins (e.g. transcription factors).

The fact that meQTL are mainly *cis*-acting and located in proximity to their related epigenetic variants, suggests that local regulatory mechanisms participate to establish the associations. A possible scenario involves distal regulative features, such as enhancer regions, whose proximity to gene promoters is exhibited through chromatin looping (**Figure 1**). Enhancer activity triggers measurable downstream events such as changes in transcriptional activity or in interconnected regulatory mechanisms, including DNA methylation. Consequently, genetic variants affecting enhancer activity, by altering the affinity of DNA binding factors (Kasowski et al., 2010) or chromatin formation (McDaniell et al., 2010), alter downstream cascades, detectable as epigenetic or eQTL. Importantly, these connections by themselves might define a functional relevance of both components, determined by a traceable chain of events. In this respect, meQTL are unlikely to represent secondary events, but rather display an important mechanism in a causal chain that provokes variability. Consistently, meQTL were suggested to present an intermediate regulator that mediates phenotypic plasticity and providing a powerful mechanism for evolutionary adaptation in changing environments (Feinberg and Irizarry, 2010).

The concept of meQTL defined by genome-scale data integration was initially introduced in two independent studies, profiling matched genotype, epitype, and gene expression information of brain tissue samples. Both studies, analyzed samples from different brain regions (Gibbs et al., 2010) and cerebellum (Zhang et al., 2010), respectively, determining genetically variable loci significantly associated to DNA methylation levels at distal gene promoters. *Ergo*, the authors suggested a functional and causal relationship between both levels of information with the genetic sequence influencing DNA methylation levels in *cis* or *trans*. Surprisingly, meQTL rarely overlapped with eQTLs, wherein transcriptional activity correlated with the underlying genotypes, suggesting meQTL and eQTLs as independent features. This observation was confirmed in studies of different tissue-types that concluded a frequent dependence of DNA methylation levels on the genetic background, however, with the majority not directly associated to expression changes (Grundberg et al., 2013). In this regard, we suggest interconnected DNA methylation and genetic sequence to provide a basal regulatory setting, poising genes and loci for activation. The actual gene activity, however, can be limited to specific cellular contexts. Consistently, a recent study integrating gene expression, DNA methylation and genotype data from three different tissue-types observed a complex relationship between the three features and questioning a strict linear cause-mediator-consequence relationship (Gutierrez-Arcelus et al., 2013).

## EPIGENETIC VARIANCE FACILITATES GWAS INTERPRETATION

In light of genome-wide association studies (GWAS), meQTL facilitate the interpretation of non-coding genetic variability and their association to phenotypic differences (Freedman et al., 2011; Hernandez and Singleton, 2012; Kilpinen and Dermitzakis, 2012). Recent studies have given an outlook of the potential of integrative genome-epigenome studies for the meaningful interpretation of genetic risk alleles (Gamazon et al., 2013; Scherf et al., 2013). Further, genotype–epitype associations guided the interpretation of physiological traits, such as natural human variation (Heyn et al., 2013) or aging (Bell et al., 2012). Moreover, in addition to guiding the interpretation of the genetic code, functional linkage could, *vice versa*, identify epigenetic driver events from often numerous differentially methylated regions (Liu et al., 2013). This symbiotic liaison highlights the power of functional linkage analysis for the meaningful mining of genetic and epigenetic variance in physiological and pathological contexts. Summarizing the current knowledge from integrative genotype–epitype analyses, the following examples also underscore the use of multidimensional datasets and their power to identify phenotype driving events.

Brain associated meQTL were shown to be implemented in neurological disease pathology and enabled the interpretation of GWAS results derived from bipolar disorders (Gamazon et al., 2013). Briefly, polymorphic risk alleles obtained from different genetic studies, revealed enrichment in *cis*-acting brain meQTL, presenting prior unrecognized disease-related genes. Importantly, incorporating *a priori* information about meQTL increased the power of detecting genotype–phenotype associations. By restricting the analysis to *cis*-acting meQTL, the authors detected a significant association between rs12618769 and bipolar disorder, which was not significant in a genome-wide screening. Intriguingly, the polymorphism was associated to differences in promoter methylation of *inositol polyphosphate phosphatase 4A* (*INPP4A*), a gene related to the functional integrity of the brain. Thus, a data set reduction to functionally connected variants enabled the identification of aberrant gene regulation with possible implication in the biology of bipolar disorders. The study presents a paradigm of how the complexity observed in genome- and epigenome-wide association studies can be reduced to loci with likely functional relevance in the given context. As initial filter steps reduce the number of required tests (which need to be corrected for in multiple hypotheses testing), functionality-driven approaches might be applicable with substantial lower sample numbers.

Following the objective to interpret GWAS, defined risk variants through epigenetic data integration, another recent study revealed differences in DNA methylation in a region previous defined as lung cancer risk locus (Scherf et al., 2013). In particular, the authors identified a significant association of the risk genotype with differential promoter methylation of the nicotinic acetylcholine receptor subunit gene *CHRNA4*, suggesting a mechanistic disease-related cascade induced by the genetic variant. Most importantly, following the validation of a direct association to transcriptional regulation, the authors confirmed the impact of increased *CHRNA4* expression on oncogenesis in lung tumor cells. Conclusively, the epigenetic activation of the *CHRNB4* correlated with the presence of genetically defined lung cancer susceptibility variants, suggesting a causal relationship between different layers of regulation in the risk allele biology.

Combining comprehensive geno- and epityping screening efforts, an adipose tissue based study detected a relationship between a metabolic disease risk loci and variant DNA methylation (Grundberg et al., 2013). Particularly, they determined the significant correlation between a SNP (rs713586) associated with body mass index and DNA methylation in an enhancer region upstream of the *adenylate cyclase 3* (*ADCY3*), a gene previously linked to obesity. Intriguingly, a SNP in perfect linkage disequilibrium directly flanking the altered CpG overlapped transcription factor binding sites, presenting potential mechanistic consequences of the genetic variant. Consistently, transcription factor binding was previously shown to influence DNA methylation levels at distal regulatory elements (Stadler et al., 2011).

## EPIGENETIC VARIANCE GUIDES INTERPRETATION OF NATURAL HUMAN VARIATION

Interestingly, the strategy of functional linkage analysis was also proven to be informative outside the disease context using variability observed between different individuals or human populations (Bell et al., 2011; Fraser et al., 2012; Heyn et al., 2013; Moen et al., 2013). Comparing three different human populations, a blood based study determined differentially methylated CpG sites between populations and confirmed the genetic blueprint to be the major factor determining epigenetic differences (Heyn et al., 2013). In particular, the study detected variance in DNA methylation between African, European, and Asian individuals with potential consequences on distinct phenotypes, including differences in drug response or susceptibility to pathogen infections. Although the variance is likely to be transmitted genetically, epigenetically defined phenotypes are exposed to the natural selection process. Accordingly, a subset of differentially methylated genes revealed evidence of local selection pressure. Most interestingly from a integrative perspective, the study determined genetic variants, associated to population-specific DNA methylation, to be enriched for SNPs previously identified as risk loci for the infection with the hepatitis B virus (HBV). Consistently, the risk SNPs were more abundant in Asian and African individuals, ethnicities for which the HBV infection presents an endemic disease. By integrating DNA methylation data, the study determined a chain of genetic and epigenetic events leading to variant *HLA-DPA1* expression, likely to drive differences in HBV infection risk between the populations.

Similarly, another study analyzing blood samples from donors with African or European ancestry determined meQTL associated with complex traits such as racial disparities (Moen et al., 2013). In particular, an association between risk alleles for cardiovascular diseases and high cholesterol levels with differential promoter methylation of the *apolipoprotein A-V* (*APOA5*) illustrated the implication of meQTL in the biology of human diseases.

Conclusively, ethnicity based studies suggested DNA methylation to be an important intermediate regulator in the translational process from geno- to phenotypes. Furthermore, it represents a valuable information to meaningful interpret variability observed between populations, however, also proving its value to explain inter-individual variation.

## GENETIC LINKAGE IDENTIFIES EPIGENETIC DRIVER EVENTS

Likely, the genetic code *vice versa* serves as an anchor point to extract functional important epigenetic variation from highly variant epigenomes. Considering the numerous epigenetic events in cancer, wherein multiple genes simultaneously gain or lose DNA methylation, the identification of epigenetic drivers presents an extremely difficult task. However, connecting DNA methylation data to additional layers of information, such as genotype or gene expression, suggests interconnected epigenetic events that are more likely to be of functional importance than unconnected alterations. In this regard, genetic connections could be of value to separate epigenetic driver from passenger events, and to simultaneously define novel risk genotypes and functional cancer genes. Herein, epitype–genotype associations represent a role-model for the interplay between different cellular mechanisms, whose interpretation will certainly provide a rich resource for disease biomarkers and strategic nodes for therapeutic intervention.

The concept of data integration for the meaningful interpretation of genome-scale DNA methylation data was successfully applied using meQTL to determine functional epigenetic events and novel genetic risk loci in rheumatoid arthritis patients (Liu et al., 2013). Specifically, the study determined differentially methylated CpG sites between patients and controls, and subsequently assessed potential genetic risk loci based on their association to aberrantly methylated CpG sites. Defining genotype as causal factor, DNA methylation as mediator and the disease as outcome, the authors assumed direct relationships between these features, which enabled the identification of genetic risk polymorphism and their underlying chain of events leading to rheumatoid arthritis. In particular, the authors defined several significant associations located within the major histocompatibility complex (MHC), genes previously related to disease risk to rheumatoid arthritis. In addition, the study identified DNA methylation levels of the *glutathione S-transferase alpha 2* (*GSTA2*) promoter region to be under genetic control. In line with the results, genetic variance in *GSTA2* family members, such as *GSTT1*, *GSTM1* and *GSTP1*, have been previously reported to predict arthritis risk and severity.

Using an integrative approach, the authors identified novel genetic risk variants. The strategy also enabled them to distinguish disease driving epigenetic events from those that might have been a consequence of the disease itself. Thus, assuming a causal relationship between the genetic and epigenetic code, functional linkage analysis is applicable for the simultaneous identification of genetic and epigenetic disease-driving events.

## INTEGRATIVE PRODUCTION PIPELINES IN INTERNATIONAL CONSORTIA

Highlighting the informative value of associations between the genotype and epigenetic regulation, this *Review* aimed to illustrate the power of data integration in the genomic era. Although presenting important information itself, genotype data only reveals its real potential after determining its interaction within the cellular context. Herein, phenotype relationships detected in GWA studies only define the endpoint in a network of intensively cross-linked cascades of events; and it is in particular their identification that enables a meaningful interpretation of significant statistical connections. Hence, considering currently ongoing massive data production efforts, we suggest a high value for the simultaneous screening of multiple layers of cellular information. In addition to providing important information for their respective research fields, their integration in multidimensional analysis pipelines will further improve meaningful readouts. Eventually, combined data from different sources of information will provide more value than the sum of its individual components.

In view of the presented examples, we encourage international multidisciplinary research consortia to continue their strategies aiming to comprehensively profile all aspects of the human genome. Although the need for simultaneous production of different data types is widely accepted and moved forward in consortia like the International Cancer Genome Consortium (ICGC, icgc.org), integrative analysis pipelines are still immature and standards still remain elusive. However, first computational tools for the integration of genotype data are developed and freely available to the broad research community (He et al., 2013; Zhang et al., 2014).

Moreover, significant associations between different layers of information need to be annotated in common portals with open access for the community. While this has been partially achieved for genotype-expression associations (He et al., 2013), novel components could amplify the information provided, in order to improve our understanding of complex cellular connections and disease biology.

The association of DNA methylation and genetic sequence displays an exemplary application, which is further extendable to virtually all traceable cellular features, including proteomics or metabolomics, among many others. It will be the knowledge of these complex relationships that will drive future efforts to resolve the conundrum of human variation in physiological and pathological contexts.

## Conflict of Interest Statement

The author declares that the research was conducted in the absence of any commercial or financial relationships that could be construed as a potential conflict of interest.
